# The influence of endothelial metabolic reprogramming on the tumor microenvironment

**DOI:** 10.1038/s41388-024-03228-5

**Published:** 2024-11-20

**Authors:** Kelby Kane, Deanna Edwards, Jin Chen

**Affiliations:** 1 Program in Cancer Biology, Vanderbilt University, Nashville, TN USA; 2 Division of Rheumatology, Department of Medicine, Vanderbilt University Medical Center, Nashville, TN USA; 3 Vanderbilt Ingram Cancer Center, Vanderbilt University Medical Center, Nashville, TN USA; 4 Department of Cell and Developmental Biology, Vanderbilt University, Nashville, TN USA; 5 Department of Veterans Affairs, Tennessee Valley Healthcare System, Nashville, TN USA

**Keywords:** Cancer microenvironment, Tumour angiogenesis, Cancer metabolism, Cancer therapy

## Abstract

Endothelial cells (ECs) that line blood vessels act as gatekeepers and shape the metabolic environment of every organ system. In normal conditions, endothelial cells are relatively quiescent with organ-specific expression signatures and metabolic profiles. In cancer, ECs are metabolically reprogrammed to promote the formation of new blood vessels to fuel tumor growth and metastasis. In addition to EC’s role on tumor cells, the tortuous tumor vasculature contributes to an immunosuppressive environment by limiting T lymphocyte infiltration and activity while also promoting the recruitment of other accessory pro-angiogenic immune cells. These elements aid in the metastatic spreading of cancer cells and contribute to therapeutic resistance. The concept of restoring a more stabilized vasculature in concert with cancer immunotherapy is emerging as a potential approach to overcoming barriers in cancer treatment. This review summarizes the metabolism of endothelial cells, their regulation of nutrient uptake and delivery, and their impact in shaping the tumor microenvironment and anti-tumor immunity. We highlight new therapeutic approaches that target the tumor vasculature and harness the immune response. Appreciating the integration of metabolic state and nutrient levels and the crosstalk among immune cells, tumor cells, and ECs in the TME may provide new avenues for therapeutic intervention.

## Introduction

**Fig. 1:**
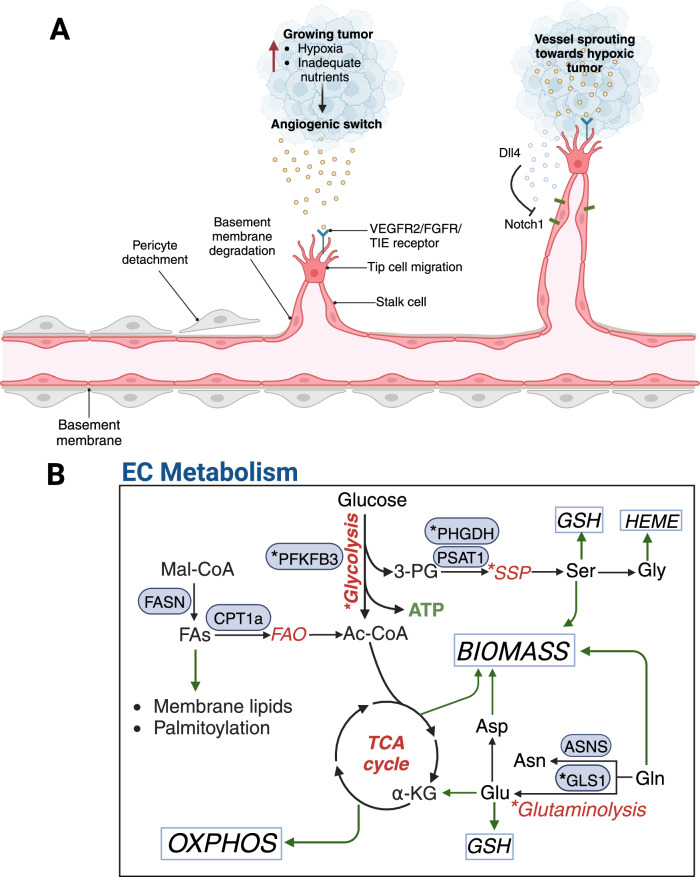
The angiogenic cascade and metabolic reprogramming of ECs in tumorigenesis. **A** Once a tumor grows to a certain size it requires an adequate supply of oxygen and nutrients from the vasculature. The hypoxic environment stimulates the release of pro-angiogenic factors that bind to receptors on endothelial cells, triggering the angiogenic switch. Pericyte detachment and basement membrane degradation facilitates endothelial tip cell migration towards the hypoxic source. Dll4 release from the tip cell binds to Notch1 on the adjacent cell to maintain the stalk cell phenotype. The imbalance of pro-angiogenic and anti-angiogenic signaling contributes to the formation of tortuous and hyperpermeable tumor blood vessels that do not function properly and further exacerbate hypoxia and nutrient deprivation. **B** Angiogenic ECs primarily utilize glycolysis for ATP production, while quiescent ECs upregulate FAO and downregulate other metabolic pathways to reduce oxidative stress. Glutaminolysis provides TCA intermediates and nitrogen for nucleotide pools to promote EC growth and migration and serves as a substrate for glutamate and asparagine production. Serine can be synthesized from the glycolytic intermediate 3-PG or taken up by ECs to support protein and nucleotide synthesis and glutathione production. In tumor endothelial cells, the glycolytic activator PFKFB3 is upregulated to drive tumor angiogenesis. Tumor ECs also display increased PHGDH expression. Glutamine and glutamine-derived aspartate are also critical metabolites supporting tumor angiogenesis. *Denotes enzymes and pathways upregulated in tumor ECs compared to ECs in normal tissues. Asn, asparagine; Asp, aspartate; Gln, glutamine; Glu, glutamate; Gly, glycine; Mal-CoA, malonyl-CoA; Ser, serine; SSP, serine synthesis pathway; TCA cycle, tricarboxylic acid cycle.

The vascular system is an extraordinary network responsible for circulating and providing the necessary nutrients and oxygen to parenchymal tissues, removing waste, and supporting immune surveillance to maintain homeostasis. Endothelial cells (ECs) line vessels and act as a barrier between tissues and blood within the vessel lumen. ECs within normal adult vasculature exist in a quiescent, non-proliferative state in most organs [1, 2]. However, the formation of new vessels, termed angiogenesis, requires a switch from a quiescent to an activated state by proangiogenic factors or hypoxia in a tightly regulated process [2]. Subsequent detachment of perivascular smooth muscle cells and breakdown of cell-cell junctions and extracellular matrix surrounding the blood vessels allows for vessel sprouting [2]. Specialized migratory ECs called tip cells use filopodia to migrate toward angiogenic growth factors like vascular endothelial growth factor A (VEGFA), while also maintaining adjacent stalk ECs through Notch1/Delta-like 4 (Dll4) signaling [1] (Fig. 1A). Angiogenesis is a critical element in tumor progression, and additional signals, including fibroblast growth factor (FGF) and FGF receptors (FGFR), angiopoietin/Tie receptor tyrosine kinases (RTKs), and ephrin/Eph receptors, also contribute to formation of dysfunctional vascular networks in tumors [3–8]. Following the discovery of the key angiogenic factor VEGFA, several antiangiogenic therapies were developed to “starve” cancer by damaging a tumor’s ability to obtain nutrients by adopting the angiogenic switch. However, despite the initial success, the clinical efficacy of these therapies was blunted by compensatory mechanisms such as the co-option of existing vessels and the induction of vasculogenic mimicry [9]. In subsequent years, strategies to target the tumor vasculature shifted away from antiangiogenics toward normalizing vessel structure and function. Several recent studies demonstrated its promise as a method to improve drug delivery and immune cell recruitment to improve tumor-related outcomes [10–12].

As a hallmark of cancer, metabolic reprogramming supports the energetic demands of cancer cells. However, other cellular components of the tumor microenvironment (TME) must also adapt their metabolism to changing conditions during tumorigenesis, including tumor-associated ECs (TECs) [13]. The advancement of single cell RNA-sequencing technology has provided insight into the vast heterogeneity of ECs regarding location and disease state, identifying metabolic genes previously unrecognized in tumor angiogenesis [14–16]. In addition to adapting their own internal metabolism, TECs also have an impact on the broader metabolic environment of the tumor by regulating the transport of essential nutrients to fuel tumor cell metabolism, which in turn modulates the fitness of infiltrating immune cells. Hypoxia and pro-angiogenic factors in the TME promote the formation of chaotic, less immune-permissive tumor blood vessels that exclude CD8^+^ T cells to infiltrate solid tumors. Notably, other immunosuppressive factors including VEGFA, transforming growth factor β (TGF-β), and adenosine can reduce the expression of adhesion molecules on the surface of ECs, compromising immune cell adherence and migration along the blood vessel wall [17]. The recruitment of other immune cells, including tumor-associated macrophages (TAMs), can support tumor angiogenesis by releasing VEGFA and metalloproteinases, which break down the extracellular matrix and release pro-angiogenic factors sequestered on the ECM [18]. In summary, the changes in oxygen and nutrient availability within the TME during the rapid tumor growth and vascularization alter the metabolic and phenotypic state of tumor cells and auxiliary cells to support growth and metastasis.

In this review, we highlight recent advances in our understanding of metabolism in vascular endothelial cells, the vascular role in nutrient delivery, and dynamic crosstalk that occurs between ECs and immune cells in the TME. We also discuss past and current anti-angiogenic therapies and spotlight more recent studies aimed at exploiting the metabolism of tumor ECs and harnessing T cells in immunotherapy as a new target against abnormal tumor vasculature.

## Metabolic reprogramming in tumor endothelial cells

### Glycolysis

Even though oxidative phosphorylation (OXPHOS) generates 34 ATP per one glucose molecule compared to net 2 ATP through glycolysis alone, ECs preferentially utilize glycolysis as their energy source (Reviewed in [19]). The transcriptome of TECs has unveiled a distinct metabolic profile compared to normal EC metabolism [14, 20] (Fig. 1B). Within the TME, TECs primarily utilize glycolysis to an even greater extent to produce ATP [21], which conserves oxygen for diffusion to the tumor and keeps reactive oxygen species (ROS) low to promote survival in hypoxic environments [19]. Angiogenic factors such as VEGFA drive a glycolytic phenotype, supporting tip cell differentiation during angiogenesis [22]. Compared to tip cells, stalk cells use less glucose primarily due to activated Notch signaling [23]. However, overexpression of 6-phosphofructo-2-kinase/fructose-2,6-biphosphatase 3 (PFKFB3), a crucial rate-limiting enzyme in glycolysis [21], can override this glycolytic suppression to promote a tip cell phenotype, supporting the driving role in angiogenesis [23].

Accumulating evidence for the role of the glycolytic activator PFKFB3 in angiogenesis and vessel barrier function further supports the notion of metabolism acting as a driving force in tumor blood vessel dysfunction [20, 24, 25]. *PFKFB3* expression is upregulated in tumor endothelial cells (TECs), and blocking the enzyme decreased melanoma metastasis and improved EC barrier integrity, independent of the presence of VEGFA [20]. Importantly, the genetic or pharmacologic inhibition of PFKFB3 promoted tumor blood vessel normalization, a newer concept in adjuvant cancer therapy that aims to restore vessel perfusion and improve chemotherapy efficacy [20]. The effects of PFKFB3 blockade extend to other cancers such as hepatocellular carcinoma (HCC) [25]. A question that arises from these findings is how in the metabolic switch PFKFB3 is regulated in angiogenesis. A study by Huang et al. uncovered a novel signaling axis by sphingosine kinase 1 (SPHK1), an enzyme that catalyzes the conversion of ceramide and sphingosine into sphingosine-1-phosphate (S1P) [26]. S1P is a bioactive lipid known to induce angiogenesis [27, 28], and SPHK1 is upregulated in certain cancers such as HCC [29]. Blocking its activity using PF-543, an inhibitor of SPHK1, attenuated angiogenesis and tumor growth in an HCC model by triggering proteasomal degradation of PFKFB3 [26]. The activity of PFKFB3 could be restored by the addition of S1P, suggesting that the levels of S1P and stability of the glycolytic enzyme are connected [26]. In the context of inflammation, TNFα increased PFKFB3 expression, where its downstream effects were modulation of NF-κB nuclear translocation and cytokine and chemokine expression to promote monocyte adhesion and migration [30]. Whether the effect of endothelial PFKFB3 overexpression could also affect monocyte recruitment in the TME requires further investigation.

The regulation of other glycolytic enzymes also plays a role in the regulation of vessel sprouting. Fructose-1,6-bisphosphatase (FBP1), the rate-limiting enzyme in gluconeogenesis and a negative regulator of glycolysis, is downregulated in hypoxia [31]. FBP1 expression is downregulated by P4HA1, the catalytic subunit of prolyl 4-hydroxylase, through Tet methylcytosine dioxygenase 2 (TET2), leading to enhanced endothelial glycolysis [31]. Further downstream of PFKFB3 and FBP1, glyceraldehyde 3-phosphate dehydrogenase (GAPDH) is elevated in TECs in human colorectal cancer (CRC) tissue compared to adjacent tissue ECs, contributing to the dysregulated tumor blood vessel phenotype [32]. Together, these studies summarize the tightly ordered regulation of key enzymes in glycolytic pathway during changes in oxygen and nutrient levels that ultimately dictate the EC phenotype.

### Fatty acid metabolism

Despite glycolysis generating ~85% of ATP for ECs, fatty acid oxidation (FAO) is also critical for normal EC function [33, 34]. It is becoming increasingly recognized the role fatty acid (FA) metabolism plays in cancer cells and now ECs [33, 35]. The uptake and FAO of long-chain-FAs (LCFAs) is mitochondrial-dependent, requiring carnitine palmitoyltransferase 1a (CPT1A) located on the outer mitochondrial membrane [36]. CPT1A facilitates FA transport into the inner mitochondrial matrix where FAO occurs [36]. In proliferating ECs, FAO sustains the pool of nucleotide precursors for deoxyribonucleotide (dNTP) synthesis to selectively support proliferation [37]. Quiescent, non-proliferative ECs upregulate FAO and concomitantly reduce glycolysis and glutaminolysis to protect against oxidative stress, since loss of *Cpt1a* increased ROS and oxidative stress in mouse ECs [38]. FAO in ECs also plays a role in endothelial barrier function, as blockade of CPT1a increased vessel permeability and leakage [39].

Endothelium metabolic rewiring is also implicated in other pathophysiological processes, such as the endothelial-to-mesenchymal transition (EndMT) that occurs in atherosclerosis or the metastatic spread of tumors [40, 41]. During this process, TGF-β signaling causes ECs to lose their cobblestone look and gain migratory and proliferative capacities, and lose characteristic endothelial “marker” genes, such as *VEGFR2*, *CDH5*, and *NOS3*. In contrast, expression of mesenchymal markers is increased, including *FSP1*, *FN1*, and *CDH1* [42]. Evidence suggests that EndMT induces metabolic changes that influence the de-differentiated state, although some controversy remains [43, 44]. A study by Xiong et al. revealed that acetyl-CoA pools and FAO are reduced early following TGF-β and IL-1β stimulation in human pulmonary microvascular endothelial cells (HPMVECs). Consequently, enhanced TGF-β signaling by SMAD2/7 modulation contributed to the EndMT phenotype, and these TGF-β mediated effects could be reversed by acetate supplementation [44]. Further, they showed that loss of CPT1a phenocopied the cytokine induced EndMT, supporting the role of FAO in gatekeeping this transition of ECs [44]. In contrast, a recent study revealed a positive feedback loop induced by TGF-β signaling that increased glycolytic flux and glucose-derived acetate, which is converted to acetyl-CoA by ACCS2. TGF-β signaling suppressed pyruvate dehydrogenase kinase 4 (PDK4), an enzyme responsible for inhibiting the actions of pyruvate dehydrogenase (PDH) producing acetyl-CoA. Increased levels of acetyl-CoA enhanced the acetylation of SMAD2/4 and ALK5, increasing their stability, where ALK5 further stimulated TGF-β signaling [43]. Importantly, supplementation with acetate induced EndMT [43]. These discrepancies warrant a more detailed investigation of the role intracellular metabolites play in maintaining normal EC phenotype. It is unclear whether these differences may be attributed to IL-1β-driven inflammation. However, the possibility remains that HUVECs/HUACs and HPMVECs, which are sourced from large and small vessels respectively, may exhibit unique metabolic responses to TGF-β stimulation.

### Amino acid metabolism

As the most abundant extracellular amino acid in the body, glutamine is taken up by cells and deaminated by glutaminase (GLS) to generate glutamate. Glutamate enters the TCA cycle as α-ketoglutarate (α-KG), the product of glutamate dehydrogenase (GDH) deamination of glutamate. The antioxidant glutathione (GSH) is also derived from glutamate and is critical for dampening ROS levels [45]. Glutamine provides carbons for ATP production in the TCA cycle as well as nitrogen for purine and pyrimidine biosynthesis [13]. ECs utilize a large amount of glutamine, and depletion of this amino acid or inhibition of GLS1 resulted in decreased vessel sprouting, proliferation, and migration of ECs [45–48]. The effects of glutamine metabolism in tumor vasculature were examined in a murine model of breast cancer [49]. EC-specific knockout of *GLS*, the rate-limiting deamination step in glutaminolysis, decreased tumor growth and metastasis, induced tumor blood vessel normalization, and enhanced chemotherapeutic delivery [49]. This finding uncovered a novel feature of tumor ECs in utilizing glutamine to promote tumor angiogenesis and provides a potential therapeutic target in tumors. Glutaminolysis-derived aspartate is also an important regulator of angiogenesis. Oberkersch et al. revealed a role of aspartate in driving mTORC1 signaling to stimulate VEGFR2 and FGFR1 expression, drive pyrimidine synthesis, and promote tumor angiogenesis [50]. These data support the critical role of amino acids in regulating EC function via direct metabolic and growth factor signaling influences.

In addition to glutamine, proliferating endothelial cells utilize the amino acid serine to support the biomass production necessary for proliferation and survival [14]. Serine can be directly taken up by amino acid transporters or de novo synthesized from glycolysis-derived 3-phosphoglycerate (3-PG). The serine synthesis pathway (SSP) is controlled by the rate-limiting enzyme phosphoglycerate dehydrogenase (PHGDH), an enzyme that is essential for EC survival and proliferation [51]. Serine feeds into the one-carbon metabolism pathway, which provides carbon for the synthesis of nucleotide precursors [13]. Glutamine-derived glutamate also serves as a critical nitrogen source necessary for de novo serine synthesis [13]. Furthermore, downstream conversion of serine by the enzyme serine hydroxyl transferase (SHMT1/2) to glycine feeds into heme synthesis. Heme is a crucial part of the prosthetic groups of cytochromes in the electron transport chain (ETC), and disruption to the ETC results in disrupted mitochondrial homeostasis and increased ROS in endothelial cells [51]. Secondary to heme maintenance, serine supports the production of GSH through cysteine biosynthesis [13].

Recent reports suggest that vascular serine metabolism may serve as an important player in cancer metastasis. PHGDH is driven by VEGFA and hypoxic stress through the transcription factor ATF4 [52]. Upregulated PHGDH in glioma TECs promoted tumor growth by production of nucleotide precursors and maintenance of redox homeostasis [52]. Furthermore, the downstream consequence of increased vascularization hampered the ability of cytotoxic T cells to infiltrate and mediate tumor cell killing. Targeting PHGDH in TECs improved CAR T cell efficacy, likely through blood vessel normalization [52]. Conversely, low expression of PHGDH correlated with increased breast cancer metastasis, and highly vascularized tumors had lower PHGDH expression [53]. Furthermore, the presence of ECs in co-culture with breast cancer cells lowered PHGDH expression, revealing a potential EC-tumor cell crosstalk that may be modulating tumor cell metabolism and thereby increasing migratory and invasive capabilities [53]. Further studies will be necessary to determine if targeting PHDGH and other metabolic enzymes may improve immunotherapy outcomes in other solid tumors. Overall, these findings highlight the influence of EC metabolism on solid tumors in contributing to tumor progression and metastasis.

## Regulation of nutrient delivery to tumors

### Signaling mechanisms governing nutrient balance

Tissue homeostasis relies heavily upon the uptake of nutrients and oxygen [54]. ECs that line blood vessels act as gatekeepers and regulate metabolite and oxygen delivery to these tissue via diffusion or directed transport mechanisms [55]. Furthermore, EC barrier tightness differs based on organ location, which can have significant impacts on nutrient access. For example, renal and pancreatic ECs are fenestrated while liver ECs are discontinuous, allowing for glucose to freely diffuse through between the ECs, respectively [55]. While the dissolved material and small molecules can cross vessel walls via ultrafiltration, glucose uptake through the GLUT family of transporters has been demonstrated to be necessary for active transport to parenchymal tissues and EC’s to meet metabolic needs [55]. Of the fourteen GLUTs in human cells, GLUT1 and GLUT3 appear to be critical in glucose uptake in ECs to support vascular function and integrity. Interestingly, GLUT3 is predominantly expressed in metabolically active tissues and tumor blood vessels, suggesting a more critical role of GLUT3 in increasing glucose uptake in these glycolytically active ECs and tumor cells [56].

Large molecules such as long-chain fatty acids (LCFA) require an assisted transport mechanism for uptake and trans-endothelial transport [35]. LCFAs are taken up by cells using specialized transporters such as the scavenger receptor CD36, fatty acid transport protein family (FATPs), and fatty acid-binding protein family (FABPs) [35]. In ECs, LCFA uptake and transport are closely coupled to mitochondrial ATP production. FATP4 sequesters LCFAs at the endoplasmic reticulum (ER) and mitochondria through its ATP-dependent acyl-CoA synthetase activity [57]. ECs also robustly express CD36 on the plasma membrane, and this transporter was necessary to maintain FA uptake within [58, 59]. It has also been demonstrated that CD36 supported loading of LCFAs into vesicular cargo in ECs, leading to exosome release and subsequent uptake by neighboring parenchymal cells [60].

**Fig. 2:**
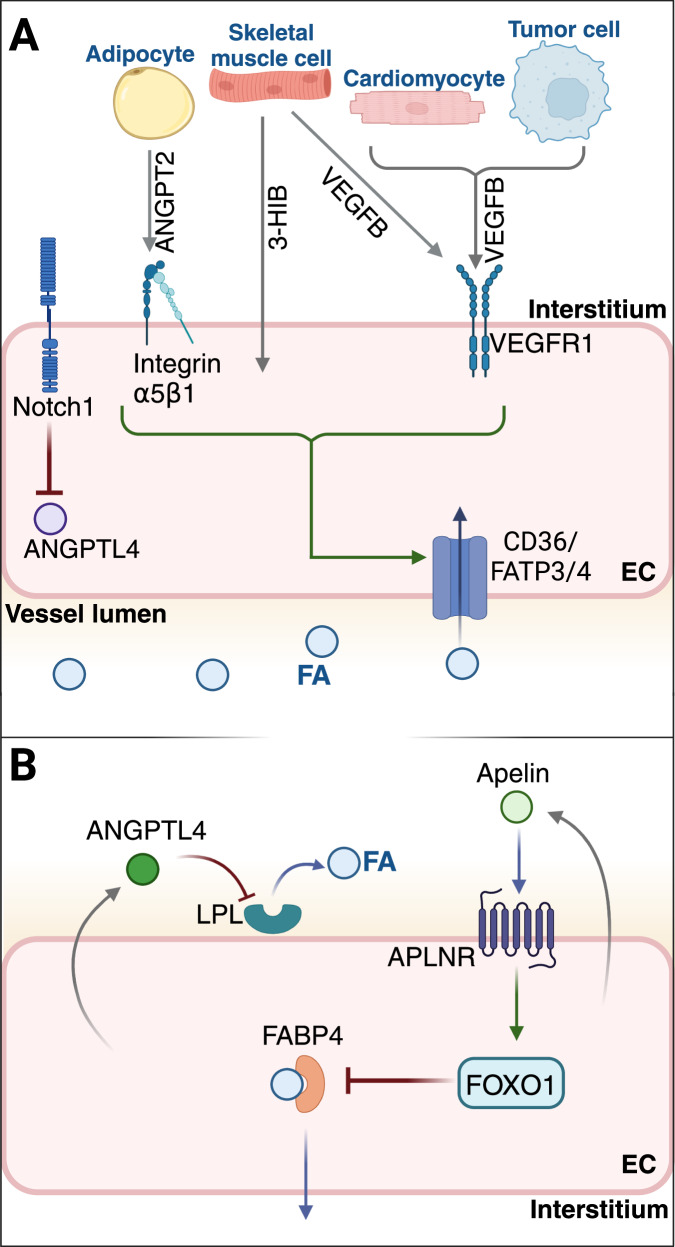
Regulation of nutrient transport across endothelial cells. ECs are gatekeepers in nutrient and oxygen delivery from blood vessel lumen to parenchymal tissues. **A** Metabolically active tissues release factors that act on ECs to stimulate FA transport. LCFA are taken up by specialized transporters such as CD36 and FATPs on the cell surface. Factors such as ANGPT2, VEGFB, 3-HIB, and Notch signaling can stimulate endothelial FA uptake. **B** Conversely, ECs negatively regulate FA transport across through different signaling mechanisms. Apelin/APLNR signaling suppresses FA uptake by activation of FOXO1 and reduces expression of its target FABP4. ANGPTL4 is pro-angiogenic by stimulating glycolysis and blocking FA uptake by suppressing LPL activity and is regulated by Notch signaling.

Parenchymal cells and ECs release several factors that regulate nutrient delivery (Fig. 2). In 2010, Hagberg and colleagues identified the growth factor VEGFB as a driver of LCFA transport [34]. VEGFB was shown to bind to VEGFR1 and its co-receptor NRP1 on the surface of ECs, leading to increased FATP3/4 expression and LCFA uptake [34]. VEGFB is more highly expressed in metabolically active tissues, such as heart and skeletal muscle, at least in part through the PPARγ coactivator 1α (PGC-1α) [61]. Metabolically active tumor cells also release VEGFB [62–64]. A recent report showed that VEGFB promoted trans-endothelial delivery of LCFAs to tumor cells through mTORC1 signaling, and loss of endothelial mTORC1 reduced LCFA in tumor cells and T cell in lung metastatic TME, leading to metastasis inhibition [65]. While mTORC1 activation was mediated through VEGFB binding to VEGFR1, it is unclear whether its co-receptor NRP1 is also involved in the signaling cascade, given its requirement for LCFA transport and endothelial function [34, 66, 67]. Active transport of LCFAs through the endothelium may be important in conditioning the pre-metastatic niche or during early metastatic outgrowth in highly vascularized organs, such as lung and liver, that are enriched in the LCFA palmitate [65, 68].

Conversely, PGC-1α has been shown to promote FA delivery to myocytes in a mechanism independent of VEGFB/VEGFR1. Instead, PGC-1α stimulated production of the branched-chain amino acid (BCAA) metabolite 3-hydroxybutyrate (3-HIB) [69]. 3-HIB secretion by myocytes promoted FA uptake of ECs and that was mainly mediated by fatty acid transporters FATP3 and FATP4 [69]. However, it is unclear how 3-HIB and BCAA metabolism may influence FA transport and uptake within tumors, particularly due to the controversial role of BCAAs in tumor biology [70]. Regardless, these studies demonstrate multiple mechanisms that stimulate endothelial FA transport (Fig. 2A).

Additional signals positively modulate nutrient delivery to tissues. The angiopoietin ligand ANGPT2 is produced by adipocytes in subcutaneous adipose tissue (SAT) and signals for transendothelial transport of FAs through binding to integrin α5β1 on ECs. Adipocyte-specific loss of *Angpt2* resulted in glucose intolerance, insulin resistance, and altered fat distribution, highlighting a novel regulator of circulating FAs [71]. Aside from ANGPT2, in vascular ECs, Notch signaling has been shown to be an important regulator of glucose and FA levels in cardiomyocytes. Loss of *Notch1* decreased expression of *CD36* and *Fabp4* along with a concomitant increase in expression of Angiopoietin-like 4 (*Angptl4*), a secreted glycoprotein that is structurally related to angiopoietins [72]. Whether these signaling mechanisms are confined to the specific tissues and if they extend to playing a role in nutrient delivery into tumors has not been thoroughly investigated.

FA transport is also negatively controlled by the ECs themselves. EC-derived ANGPTL4 promoted tumor angiogenesis by maintaining a glycolytic phenotype and inhibiting lipoprotein lipase (LPL) [73]. LPL is responsible for hydrolyzing triglyceride-rich lipoproteins in the bloodstream for FA transportation across ECs to neighboring tissues and its inhibition led to suppressed FA delivery [74]. In contrast, EC-specific deletion of *Angptl4* increased FA uptake and rendered vessels quiescent and stable, highlighting a relationship between ECs regulating lipid uptake and the effects on intrinsic metabolic state [73]. Similarly, the ligand apelin and its cognate receptor APLNR expressed on ECs regulate the distribution of glucose and lipids to metabolically active tissues such as adipose tissue and skeletal muscle in adults [75]. Apelin-APLNR signaling on ECs suppressed FA uptake by activation of transcription factor FOXO1 and reduced expression of downstream FA-binding protein FABP4 [75]. Consequently, loss of apelin resulted in ectopic lipid accumulation and glucose intolerance, supporting the notion of reciprocal regulation between glucose and FA levels between adjacent tissues [75]. Collectively, these singular findings suggest multiple signaling mechanisms by which ECs intrinsically modulate FA transendothelial transport to parenchymal cells (Fig. 2B).

### Potential impact of dynamic palmitoylation coordinates nutrient uptake

An interesting area of research that stems from nutrient uptake is the regulation of these specialized transporters at the cell surface by post-translational modifications (PTM). The FA palmitate can be added to proteins, typically on cysteine residues, to modulate protein trafficking or function [76]. Palmitoylation is reversible, allowing for dynamic cycling of the palmitoylated and unmodified states in response to environmental cues [77]. These dynamics are regulated by zinc-finger DHHC-containing palmitoyl acyltransferases (ZDHHCs) and acyl protein thioesterases (APT1/2), which add or remove the palmitate modifications, respectively [76]. Palmitoylation was recently shown to regulate CD36-mediated uptake of FAs in adipocytes, where FA uptake and CD36 internalization inactivated ZDHHC5, allowing CD36 to be de-palmitoylated by APT1 [78]. The subsequent recruitment of SYK, JNK, and VAV promoted endocytic uptake and delivery of FAs for storage in lipid droplets [78]. In glioblastoma cells, palmitoylation of GLUT1 was also necessary for its localization at the cell surface to sustain the glucose supply and glycolysis [79]. The impact of this PTM on other solid tumors has not yet been elucidated, though it could be that dynamic palmitoylation activity varies across different tumor types depending on organ location and the metabolic dependencies of the tumor.

It is currently unclear whether palmitoylation regulates nutrient uptake and trafficking in endothelial cells. However, novel palmitoylated targets that regulate VEGFA, or insulin-induced angiogenesis have been uncovered [80]. Furthermore, hyperglycemia and elevated free FAs associated with diabetes altered the palmitoylation state and activity of APT1, resulting in disrupted endothelial cell membrane dynamics and vessel integrity [81]. Due to the importance of CD36 and FA uptake in ECs for delivery to parenchymal tissues, it is enticing to speculate that palmitoylation cycling could also be implicated in FA uptake and transport across ECs. Additionally, the altered metabolite states within tumors may have significant effects on post-translational modification of TECs, adding another layer of complexity to vascular function.

## Impact of vascular endothelium on antitumor metabolic immune microenvironment

Tumor cells exhibit enhanced flux of glycolysis and glutaminolysis to support their proliferation and progression [82]. Dysfunctional vasculature within tumors further exacerbates metabolic stress within tumors, due to inadequate delivery of nutrients and oxygen and inefficient removal of metabolic waste. Lactate accumulation resulting from tumor cell glycolysis has shown dual effects on ECs and immune cells via increased angiogenesis and immunosuppression [83]. The presence of other microenvironmental cells with distinct metabolic programs also creates a metabolic environment that lends itself to immune evasion and tumor progression [82]. For example, glutamine competition between glutamine-addicted breast cancer cells and anti-tumor T lymphocytes impairs T cell activity and targeting GLS in tumor cells reduced tumor growth and metastasis and improved T cell activation [49, 84]. It is important to note that the tumor type and organ site introduce unique metabolic environments that can have significant impacts on nutrient availability [85]. Therefore, vascular nutrient maintenance may have varying consequences on tumor immune function and contribute to immune evasion.

Impaired nutrient delivery and poor oxygen supply creates hypoxic regions that promote an immunosuppressive environment. Hypoxia induces the expression of PD-L1 on TECs and tumor cells, which in turn blocks CD8^+^ T cell activation and proliferation [86, 87]. Hypoxia-induced expression of VEGFA potentiates the ability of interleukin-10 (IL-10) and prostaglandin-E2 (PGE2) to drive expression of Fas ligand (FasL) on tumor ECs to selectively induce apoptosis of cytotoxic T cells but leave T_reg_ cells unscathed [88]. Further, T_reg_ cells are recruited in response to tumor hypoxia and are producers of VEGFA to promote angiogenesis and further support an immunosuppressive TME [89].

While the endothelium can directly suppress T cell activity through PD-L1 expression or creation of an unfavorable TME [86], the tumor vasculature can also limit T cell accessibility [90]. Excessive angiogenesis forms immature and dysfunctional blood vessels that limits cytotoxic T cell ingress, transitioning tumors from immunologically “hot” to “cold” as the ability for CD8^+^ T cells to be trafficked and infiltrate the tumor and are restricted to the tumor periphery or not present at all, creating challenges in the clinical setting [91, 92]. Current cancer treatments such as chemotherapy and immunotherapy can exhibit limited efficacy in patients in part due to the dysfunctional vasculature and thus induce therapeutic resistance and poor outcomes [91]. Recent strategies to promote vessel normalization have been described, and these have demonstrated improvements in lymphocyte infiltration and function. For example, targeting endoglin, a protein involved in vascular remodeling and angiogenesis, promoted tumor blood vessel normalization, increased T lymphocyte and NK cell infiltration and repolarized TAMs to an anti-tumor state in melanoma [93]. Loss of mTORC1 signaling in endothelial cells through deletion of the subunit *Raptor* or use of a low dose of the mTORC1 inhibitor everolimus also normalized tumor blood vessels and increased immune cell recruitment and adoptive T cell transfer [94]. Furthermore, tumor vasculature and anti-tumor immunity could have a reciprocal relationship in vessel normalization. Indeed, T helper (Th1) CD4^+^ T cells positively modulate blood vessel integrity through secretion of IFN-γ and enhanced T lymphocyte infiltration [95]. These findings support the approach of promoting vessel normalization in conjunction with immune checkpoint blockade (ICB) to improve immune cell infiltration.

Active transport of LCFA across ECs, at least in part through VEGFB-VEGFR1 signaling, fuels tumor cell proliferation while also influencing anti-tumor T cell function [65, 96–98]. While LCFA supports the effector function of CD8^+^ T cells in some cases, accumulation of some LCFAs can lead to diminished function [99]. It has been demonstrated that in the presence of palmitate, CD8^+^ T cells underwent transcriptional changes that reduced their ability to store and safely utilize FAs, leading to a diminished ability to produce anti-tumor factors including IFNγ, TNFα, and granzyme B, reducing their effector function [96–98, 100]. Elevated levels of LCFA also enhanced lipid peroxidation and ferroptosis of CD8^+^ T cells, preventing sustained anti-tumor responses [97, 98]. Thus, preventing FA accumulation in the TME by targeting EC-mediated transport could potentially restore CD8^+^ T cell response. Additionally, while normalizing tumor blood vessels enhances recruitment of CD8^+^ T cells, simultaneous improved nutrient transport could support tumor growth and negatively impact T cell function. The dual role of lipids in the TME serving pro- and anti-tumor effects still lacks full elucidation and requires more mechanistic insight. Moreover, different organ metabolic microenvironments may have varying lipid species that could also support the metabolic function of other immunosuppressive cells such as TAMs, which have been shown to require FAs to drive their polarization [101]. The presence of these cells in the TME could in turn suppress T cell activation and proliferation.

In addition to nutrient and oxygen depleted conditions, the metabolic state of TECs drives tumor angiogenesis and poses significant effects on immune cells within the TME [20, 49]. Immune populations in the microenvironment are in close association with tumor cells and blood vessels. TAMs are a dominant cell type in the TME and contribute to angiogenesis and metastatic burden in solid tumors [102–104]. In support of this, TAMs played an intermediary role between invading breast tumor cells and the endothelium by priming the vascular niche. Here metastasizing tumor cells activated macrophages whereby they triggered an inflammatory response in ECs to enhance tumor cell stemness and viability at the lung metastatic site [105].

The mTORC1 pathway senses environmental signals, including nutrient availability, to regulate cellular growth, proliferation, migration, and metabolism [106]. In TAMs, mTORC1 plays a significant role in cellular interactions. While low mTORC1 activity supported localization near tumor cells, hyperactivation of mTORC1 via loss of the negative regulator TSC1 shifted their localization to perivascular regions. The effects of this change in TAM localization within the tumor were two-fold: (1) *Tsc-1* null TAMs outcompeted a subset of ECs known as endothelial progenitor cells to block tumor angiogenesis and (2) induced tumor hypoxia and cancer cell death [103]. The evolving tumor vasculature also increases the emergence of hypoxic niches, which have been shown to recruit and entrap TAMs away from perivascular regions [102]. These sequestered TAMs display a distinct gene signature that contributes to immunosuppression of neighboring cytotoxic CD8^+^ T cells [102] and blood vessel hyperpermeability [104]. Leveraging the mTORC1 pathway could be one approach in targeting the altered nutrient and metabolic states of cells in the TME, but secondary impacts of blocking mTORC1 in immune cells should be carefully considered as its effects have been shown to be both anti-tumor and immunosuppressive [82].

Targeting hypoxic TAMs could promote vessel normalization and be used in conjunction with other cancer therapies to enhance drug delivery [104]. Hypoxic TAMs are highly glycolytic and can compete for glucose with TECs and other cells within the TME [107]. Blocking REDD-1 (regulated in development and DNA damage response 1), a negative regulator of mTORC1, further increased glycolysis in TAMs and limited glucose availability to TECs [107]. In support of the driving role of metabolic state modulating EC activation and angiogenesis, decreased glycolytic flux in the TECs led to endothelial quiescence and vessel normalization [107]. Together, these findings underpin a close yet ever-evolving relationship that occurs between macrophages and the endothelium where macrophages initially prime the vascular niche to facilitate tumor progression yet continue to co-exist and influence the same niche as the metabolic state of the TME evolve.

## Harnessing tumor blood vessel normalization with immunotherapy

**Fig. 3:**
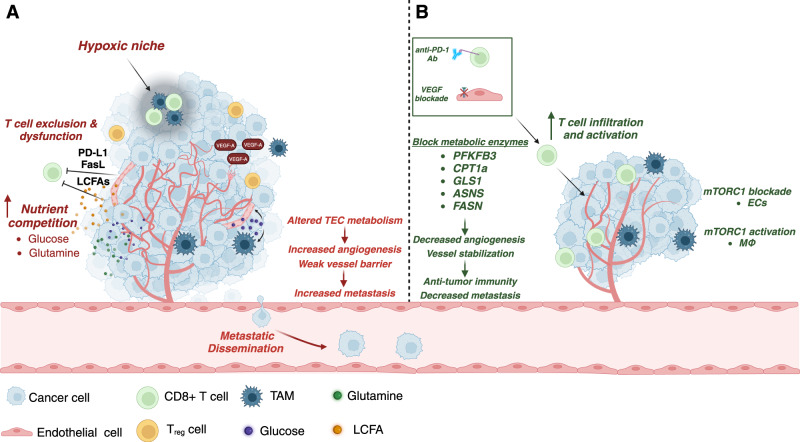
Interactions between immune cells and tumor endothelial cells in the tumor microenvironment. The TME composition varies between cancer type and organ site. **A** The release of VEGFA from tumors and other cell types in the TME such as T_reg_ cells and TAMs contributes to excessive angiogenesis and the formation of immature blood vessels that are unable to effectively deliver oxygen and nutrients and remove waste within the TME. This further exacerbates the harsh conditions as metabolites such as glucose and glutamine are scarce, which are critical for the function of cells involved in anti-tumor immunity such as CD8^+^ T cells. Altered glucose and changes in nutrient sensing signaling pathways such as mTORC1 also affect the localization of cells within the TME, and the formation of hypoxic niches sequester TAMs and CD8^+^ T cells. Besides poor infiltration, TECs also contribute to immunosuppression by expression of ligands PD-L1 and FasL, which contribute to T cell dysfunction. Intrinsic TEC metabolism also contributes to angiogenesis and poor barrier integrity, promoting tumor cell intravasation and metastasis. Lastly, uptake of LCFAs by CD8^+^ T cells have immunosuppressive effects, and it was recently shown that VEGFB-mediated delivery of LCFAs across ECs has tumor-promoting effects in the lungs. **B** Targeting angiogenesis with traditional VEGF/VEGFR2 inhibitors in combination with immunotherapy has synergistic effects as tumor vessel normalization facilitates T cell infiltration and tumor killing abilities, which in turn reduces the feedback loop of excessive angiogenesis from tumor cell-derived pro-angiogenic factors. Additionally, targeting EC metabolism in pathological angiogenesis could also be a potential treatment approach for targeting tumor vessel normalization and improved immunotherapy efficacy and decreased metastasis.

The first anti-angiogenic therapies targeting VEGFA (bevacizumab) and RTK (sunitinib, axitinib, and sorafenib) initially showed promise but did not increase overall patient survival. Unfortunately, the desired effects of starving tumor cells by cutting off their blood supply resulted in a rebound effect due to increased hypoxia and compensatory upregulation of other pro-angiogenic regulators. Additionally, loss of blood vessel flow decreases infiltration of immune cells that exert their antitumor function [108]. Overall, these pose challenges in cancer treatment, but normalization of the tumor vasculature has been identified as a new strategy to increase vessel stabilization and perfusion (Fig. 3). Altering the metabolic state of tumor ECs driving angiogenesis and barrier function could lead to vessel normalization, offering the benefit of bypassing angiogenic receptor signaling to improve cancer therapy (Table 1).

**Table 1:** Selected inhibitors targeting glucose, fatty acid, and amino acid metabolism as potential targets in tumor angiogenesis.

Metabolite	Target	Drug	Study status	Indication (s)	Notes	References (ClinicalTrials.gov)
Glucose	PFKFB3	3PO	Preclinical	Melanoma	In combination with cisplatin	[20]
		PFK-158	Phase l	Advanced solid tumors	Drug tested on multiple cancer cell lines	[118]
Fatty acids	CPT1	Etomoxir	Preclinical	Pathological ocular neovascularization	Not tested in tumor angiogenesis	[37]
	FASN	Orlistat	Preclinical	Pathological ocular neovascularization	Blocked mTORC1 activation through malonlylation	[113]
Glutamine	GLS1	CB-839	Preclinical	Breast cancer	In combination with cisplatin	[49]
			Phase I	RCC	In combination with cabozantibib (VEGFR2 inhibitor)	NCT03428217
			Phase I	Advanced solid tumors	Subset of patients had increased PFS	NCT02861300 [119]
			Phase I	RCC	Treatment demonstrated efficacy in subset of patients	NCT02071862 [109]
Serine	PHGDH	NCT503	Preclinical	Glioblastoma	In combination with CAR T cell therapy	

*RCC* renal cell carcinoma.

Inhibitors targeting various metabolic enzymes have shown efficacy in pre-clinical models, and some have entered clinical trials. Blocking the glycolytic activator PFKFB3 using the small molecule 3-(3-pyridinyl)-1-(4-pyridinyl)-2-propen-1-one (3PO) decreased vessel leakiness, improved perfusion, and promoted vessel normalization in a melanoma mouse model [20], and reduced EC growth in vitro and in vivo in a pathological ocular angiogenesis model [24]. Due to the importance of serine synthesis and metabolism in endothelial cell dynamics, pharmacological inhibition of PHGDH using NCT503 or WQ2201 abrogated tumor vessel sprouting and enhanced sensitization to CAR-T therapy in glioblastoma [52]. Blocking glutamine metabolism in ECs through the GLS1 inhibitor telaglenastat (or CB-839) also reduced EC sprouting and proliferation [45, 49], and enhanced chemotherapeutic efficacy when used in combination with cisplatin [49]. CB-839 is also being tested in phase I and ll clinical trials for various solid cancers [109–111]. Since glutamine also serves as a source for asparagine, blocking ASNS could show potential in targeting EC metabolism, though the inhibitors blocking this enzyme have not been studied in the context of modulating the tumor vasculature [112]. While little is known regarding the vascular impact of targeting FA metabolism in tumor models, the use of etomoxir, a CPT1a inhibitor, has shown some benefit in an ocular retinopathy model [37]. However, blocking FA synthesis using the FASN inhibitor orlistat has anti-angiogenic properties [113]. These studies highlight the exciting potential of targeting metabolism to improve vascular function in tumors, possibly in combination with antitumor drugs or immunotherapy to improve cancer outcomes.

The reciprocal interactions between immune cells and TECs also offer the potential of combining traditional angiogenic inhibitors with ICB. Already, clinical trials have been completed or are underway and show promise in overcoming some barriers to cancer treatment (Table 2), and many have shown improved clinical responses. The treatment of bevacizumab and PD-L1 monoclonal antibody atezolizumab showed elevated CD8^+^ T cell activation markers and improved T cell migration in metastatic renal cell carcinoma [114] and improved patient outcomes in HCC [115]. More recently, a phase II clinical trial evaluating the combination of anti-PD-1 monoclonal antibody with the histone deacetylase inhibitor chidamide and anti-VEGFA therapy, showed that the addition of anti-angiogenic therapy increased CD8^+^ T cell infiltration in patients with colorectal cancer patients compared to those treated with anti-PD-1 and chidamide alone [116]. In addition to the impacts on normalizing vessel structure and function, targeting metabolism may have further benefits when combined with ICB therapies, such as reduced PD-L1 expression by tumor cells and endothelial cells when targeting glycolysis [86, 87]. These trials demonstrate the promise of including vessel-targeting therapies in combinatorial treatment regimens to improve immunotherapy outcomes.

**Table 2:** Ongoing and completed clinical trials of combined anti-angiogenic treatment and immunotherapy.

Inhibitor	Clinical stage	Study status	Indication(s)	Notes	References (ClinicalTrials.gov)
Axitinib	lll	Completed; **FDA approved**	RCC	In combination with PD-1 blocker and chemotherapy	NCT02684006 [120] • Prolonged PFS NCT02853331 [121] • Improved OS, PFS
Bevacizumab	ll	Completed	Colorectal liver metastases	In combination with ICI	NCT03698461 • Higher response with combination therapy than anti-PD1 alone. Higher PFS and OS
	II	Completed	Colorectal cancer	In combination with PD-1 blocker and HDACi	NCT04724239 [116] • Enhanced CD8^+^ T cell infiltration. Longer PFS and overall response rate.
	lI	Completed	Metastatic colorectal cancer	In combination with ICI and MAPK inhibitor	NCT02876224 [122] • Acceptable safety profile, trend for increased benefit in patients with RAS mutations compared to those without.
	l/ll	Completed	Metastatic solid tumors	In combination with NK immunotherapy	NCT02857920 • No outcome reported
	lll	Completed; **FDA approved**	NSCLC	In combination with ICI and chemotherapy	NCT02366143 [123] • OS improvement
	lll	Completed; **FDA approved**	Ovarian cancer	In combination with PARP inhibitor	NCT03737643 [124] • Improved PFS
	ll	Recruiting	HCC	In combination with a PD-L1 blocker and ICI	NCT03937830
	ll	Recruiting	Breast cancer	In combination with ICI and chemotherapy	NCT04739670
	ll	Recruiting	NSCLC	In combination with PD-L1 blocker and chemotherapy	NCT05738317
	I/II	Ongoing	Ovarian cancer	In combination with chemotherapies	NCT04938583
Cabosantinib	III	Completed; **FDA approved**	RCC	In combination with ICI	NCT03141177 [125] • Improved OS and ORR
Ramucirumab	ll	Recruiting	NSLC	In combination with ICI	NCT03527108
Pazopanib	l/ll	Recruiting	Refractory solid tumors	In combination with PD-1 blocker	NCT05210413
	lll	Recruiting	NSCLC	In combination with PD-1 blocker	NCT05633602
	ll	Recruiting	Gastric cancer	In combination with PD-1 blocker and chemotherapy	NCT04069273

*HCC* hepatocellular carcinoma, *NSCLC* non-small cell lung cancer, *ICI* immune checkpoint inhibitor, *TKI* tyrosine kinase inhibitor, *HDACi* histone deacetylase inhibitor, *PFS* progression-free survival, *OS* overall survival, *ORR* overall response rate.

## Conclusions and future perspectives

The metabolism of ECs that line blood vessels has only recently been considered as a critical factor in regulating angiogenesis and vessel function. These cells act as gatekeepers in the delivery of nutrients and oxygen to metabolically active tissues. The well-established notion that once a tumor reaches 1−2 mm it must generate its blood supply, for nutrients and oxygen, now extends to examining the reciprocal regulation between tumor cells, ECs, and immune cells [3]. The growing tumor leads to two major problems: increasing oxygen deprivation and abnormal blood vessel formation. These issues create an environment where cells struggle to get necessary nutrients and tumor blood vessels cannot effectively remove waste products. The altered metabolite and waste levels can exert either supportive or detrimental effects within the TME, depending on cell type, that ultimately support tumor progression. Furthermore, it is not well understood how organ-specific morphology and features of blood vessels and ECs together with the organ-specific metabolic environments could also affect metastasis patterns. The uptake of nutrients and metabolic demands of cells in the TME are tightly linked to the state of ECs yet are studied independently of each other. Adding to the complexity is the role of protein lipidation such as palmitoylation in regulating nutrient uptake and vessel integrity which underlies the importance of studying these in parallel with one another.

Comprehensive analyses of TECs across multiple cancer types have identified features of different EC subsets within the tumor and potential interactions with other cells within the TME that could provide insight into predicting patient prognosis and response to anti-angiogenic therapies [117]. These studies generate hypotheses to investigate specific signaling pathways regulating the crosstalk between the endothelium in the TME with tumor cells and immune cells in different organ sites of primary tumors and metastases. TECs are closely coupled with chemotherapeutic and immunotherapy effectiveness and resistance, as the ability of the drug to infiltrate the tumor and overcome the immunosuppressive and metabolic barriers is largely dependent on the state of the tumor vasculature. How crosstalk among ECs with tumor cells and immune cells could be targeted to treat cancer and prevent metastases without compromising normal function of these cells is of great importance in developing effective treatment strategies. The most common cause of cancer-related death is metastases, so it is necessary to delineate the metabolic and location-specific heterogeneity of tumor vasculature and its role in tumor progression. Answering these questions will open exciting avenues for targeting the metabolic state of TECs to improve immunotherapies and traditional cancer therapies.
